# Suicide candidate genes associated with bipolar disorder and schizophrenia: An exploratory gene expression profiling analysis of post-mortem prefrontal cortex

**DOI:** 10.1186/1471-2164-8-413

**Published:** 2007-11-12

**Authors:** Sanghyeon Kim, Kwong-Ho Choi, Ali Fuat Baykiz, Howard K Gershenfeld

**Affiliations:** 1 Department of Psychiatry, Univ. of Texas Southwestern Medical Center, Dallas, Texas 75390-9070, USA; 2 Stanley Foundation Laboratory of Brain Research, Department of Psychiatry, Uniformed Services University of Health Sciences, Bethesda, MD 20892, USA; 3 Elazig Asker Hastanesi Psikiyatri Klinigi 23300 Elazig, Turkey; 4 *Department Integrative Biology, Univ. of Texas Southwestern Medical Center, Dallas, Texas 75390-9070, USA

## Abstract

**Background:**

Suicide is an important and potentially preventable consequence of serious mental disorders of unknown etiology. Gene expression profiling technology provides an unbiased approach to identifying candidate genes for mental disorders. Microarray studies with post-mortem prefrontal cortex (Brodmann's Area 46/10) tissue require larger sample sizes. This study poses the question: to what extent are differentially expressed genes for suicide a diagnostic specific set of genes (bipolar disorder vs. schizophrenia) vs. a shared common pathway?

**Results:**

In a reanalysis of a large set of Affymetrix Human Genome U133A microarray data, gene expression levels were compared between suicide completers vs. non-suicide groups within a diagnostic group, namely Bipolar disorder (N = 45; 22 suicide completers; 23 non-suicide) or Schizophrenia (N = 45; 10 suicide completers ; 35 non-suicide). Among bipolar samples, 13 genes were found and among schizophrenia samples, 70 genes were found as differentially expressed. Two genes, *PLSCR4 *(phospholipid scramblase 4) and *EMX2 *(empty spiracles homolog 2 (Drosophila)) were differentially expressed in suicide groups of both diagnostic groups by microarray analysis. By qRT-PCR, *PLSCR4 *and *EMX2 *were significantly down-regulated in the schizophrenia suicide completers, but could not be confirmed in bipolar disorder.

**Conclusion:**

This molecular level analysis suggests that diagnostic specific genes predominate to shared genes in common among suicide vs. non-suicide groups. These differentially expressed, candidate genes are neural correlates of suicide, not necessarily causal. While suicide is a complex endpoint with many pathways, these candidate genes provide entry points for future studies of molecular mechanisms and genetic association studies to test causality.

## Background

Suicide is the eleventh leading cause of death for all Americans with an age-adjusted annual rate of 10.5 per 100,000 in 2003 [1]. More than 90% of suicide completers have a psychiatric disorder and mood related disorders are the most common disease associated with suicide [2,3]. Patients suffering with bipolar disorder and schizophrenia have greatly increased rates of suicide with approximately 10% of patients dying of suicide [4,5]. Bipolar disorder and schizophrenia share common risk factors for suicide completion such as depression, previous suicide attempts, hopelessness, substance abuse, agitation, and poor adherence to treatment [4,5]. Suicide is a complex endpoint with many factors and pathways leading to death [6]. The hypothesis of a shared causation for suicide suggests common pathways and genes may function as susceptibility factors in both disorders. Alternatively, there could be specific distinct pathways within a diagnostic group.

Microarray technology provides an unbiased approach to the molecular causes of psychiatric disorders by examining the gene expression profile of cases vs. controls [7,8]. Recent microarray studies identified differentially expressed genes between suicide and depression patients vs. normal controls [9-11]. However, due to the small magnitude of the differential gene expression, the genetic heterogeneity of these mental disorders, and the mixed cellular nature of the brain tissue available [12], microarray studies with small sample sizes are prone to generate many false positive results [13]. Analysis of larger data sets pooled from independent studies increase the statistical power to find differentially expressed genes with small effect sizes in microarray studies. Recently, a large microarray data set generated by the Stanley Medical Research Institute has become available online (SMRIDB) [14]. This database contains clinical information and microarray data from 12 independent studies with post-mortem brain tissues of depression, bipolar disorder, schizophrenia, and unaffected control cohorts [15]. In this study, we reanalyzed this large microarray data set of bipolar disorder and schizophrenia patients. The question posed is, "to what extent are differentially expressed genes for suicide specific to diagnosis (bipolar disorder vs. schizophrenia) vs. a shared common pathway?"

## Results

### Suicide candidate genes in bipolar disorder and schizophrenia

Chi square tests of association indicated no difference in demographic variables between suicide vs. non-suicide subgroups within bipolar disorder. In contrast, age and smoking showed significant differences with suicide vs. non-suicide in the schizophrenia subgroup. Also, brain pH and sex significantly affected the expression levels of the differentially expressed genes between suicide vs. non-suicide groups within schizophrenia (Table 1). As shown in Table 1, none of these variables met the two criteria for inclusion as covariates in the two disorders (see methods). Therefore, we used no covariates in the omnibus model to provide a generalizable, single, same model for both schizophrenia and bipolar disorder. Between the suicide vs. non-suicide groups within bipolar disorder, a total of 13 genes were differentially expressed (Table 2). Among these genes, 10 genes were down-regulated and 3 genes including gamma-amino butyric acid A receptor, α5 subunit (*GABRA5*) were up-regulated.

**Table 1:** Demographic factors of the suicide group and the non-suicide group in bipolar disorder and schizophrenia

	Bipolar disorder (n = 45)				Schizophrenia (n = 45)			
	Suicide n = 22	Non-suicide n = 23	vs^1 ^suicide	vs^2 ^expression	Suicide n = 10	Non-suicide n = 35	vs^1 ^suicide	vs^2 ^expression

Age	44.3 ± 10.9	45.4 ± 11.3	n.s	n.s	34.5 ± 7.6	44.3 ± 9.1	0.003	n.s
Sex (M/F)	11/11	12/11	n.s	n.s	6/4	28/7	n.s	< 0.001
PMI	36.8 ± 19.3	36.8 ± 16.9	n.s	n.s	35.3 ± 17.9	31.4 ± 15.0	n.s	n.s
Brain pH	6.4 ± 0.3	6.4 ± 0.3	n.s	< 0.0001	6.4 ± 0.3	6.4 ± 0.3	n.s	0.005
Smoking^3^	8/9/5	13/6/4	n.s	n.s	3/2/5	25/5/5	0.03	n.s
Alcohol ^4^	2/7/4/2/5/2/0	4/4/2/3/5/4/1	n.s	n.s	3/2/1/0/2/2	9/7/4/4/3/8	n.s	n.s
Drug abuse^5^	7/3/4/4/1/3	9/1/2/2/2/7	n.s	n.s	4/1/1/0/2/2/0	17/3/2/3/3/5/2	n.s	n.s

^1^P-values less than 0.05 were considered significant.

^2^P-values listed represent the lowest. FDR P-values less than 0.05 were considered significant.

^3^Smoking; Yes/No/Unknown

^4^Alcohol; Little or none/Social/Moderate drinking in past/Moderate drinking in present/Heavy drinking in past/Heavy drinking in present.

^5^Drug abuse; Little or none/Social/Moderate drug use in past/Moderate drug use in present/Heavy drug use in past/Heavy drug use in present/Unknown.

The continuous variables represent the mean ± S.D. n.s., not significant.

**Table 2:** Summary of differentially expressed genes between the suicide completer group vs. non-suicide group in bipolar disorder with fold change relative to non-suicide group.

**Gene symbol**	**Description**	**FC**^1^	**P-value**^2^	**P/A call**^3^
TM4SF1	transmembrane 4 L six family member 1	-1.6	0.07	P
CHI3L1	chitinase 3-like 1 (cartilage glycoprotein-39)	-1.5	0.05	P
EMX2	empty spiracles homolog 2 (Drosophila)	-1.4	0.09	P
LDLR	low density lipoprotein receptor	-1.4	0.05	P
ZIC1	Zic family member 1 (odd-paired homolog, Drosophila)	-1.4	0.05	P
PLSCR4	phospholipid scramblase 4	-1.4	0.05	P
ZHX2	zinc fingers and homeoboxes 2	-1.4	0.07	P
	MRNA; cDNA DKFZp586B211 (from clone DKFZp586B211)	-1.4	0.09	P
TIMP1	TIMP metallopeptidase inhibitor 1	-1.3	0.05	A
NAV2	neuron navigator 2	-1.3	0.05	P
TRIM23	tripartite motif-containing 23	1.4	0.09	P
STCH	stress 70 protein chaperone, microsome-associated, 60 kDa	1.3	0.09	P
GABRA5	gamma-aminobutyric acid (GABA) A receptor, alpha 5	1.3	0.09	P

^1^FC represents ratio of geometric means between suicides vs. non-suicide.

^2 ^P-values were adjusted by FDR.

^3^Absent (A) or Present (P) call by MAS 5

**Figure 1:**
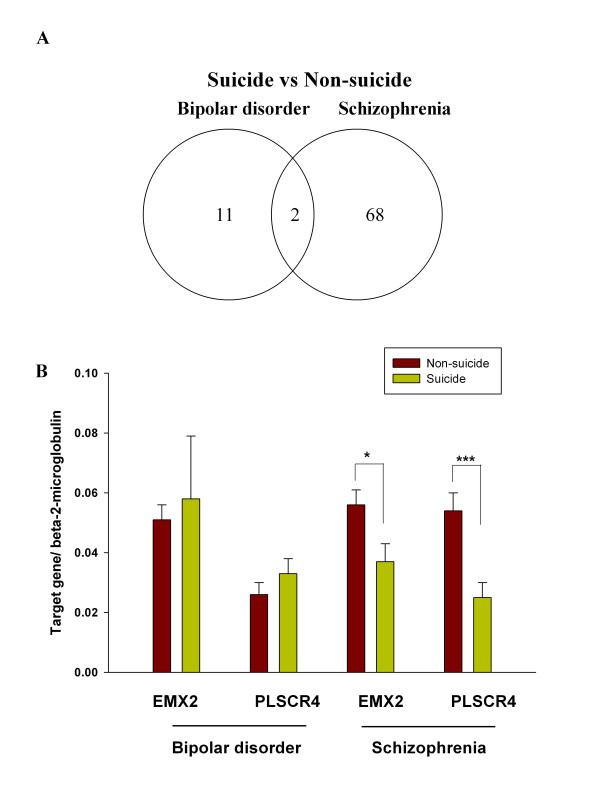
**Differentially expressed genes between suicide completer vs. non-suicide group in bipolar disorder and schizophrenia cohorts**. (A) Venn diagram of differentially expressed genes between suicide completer vs. non-suicide groups within bipolar and schizophrenia. (B) Mean expression levels of *PLSCR4 *(phospholipid scramblase 4) and *EMX2 *(empty spiracles homolog 2 (Drosophila)) mRNA transcripts were determined for suicide completers vs. non-suicide groups within both bipolar disorder and schizophrenia in prefrontal cortex by qRT-PCR. The bars represent mean ± SEM. In schizophrenia, the mean expression levels of both genes were significantly down-regulated in the suicide group (n = 5) relative to the non-suicide cases (n = 25) by one-tailed, t-tests for unequal variances (*EMX2 *t(9) = 2.42, p = 0.02; *PLSCR4 *t(18) = 3.77, p = 0.0005). The estimated fold changes in the suicide group were -1.51 for *EMX2 *and -2.16 for *PLSCR4 *relative to the non-suicide group, consistent with our microarray data. In the bipolar disorder samples, no significant differences in mean expression levels for either gene were found between the suicide (n = 14 for *EMX2*; n = 12 for *PLSCR4*) vs. non-suicide cases (n = 15 for *EMX2 *; n = 11 for *PLSCR4*) by unequal variance t-tests. * p < .05 ; *** p < .001.

Between the suicide vs. non-suicide group within schizophrenia, 70 genes were differentially expressed (Table 3). Most of these genes were down-regulated. From the above lists of differentially expressed genes, within diagnostic groups, two genes overlapped (Fig. 1A). Specifically, the phospholipid scramblase 4 (*PLSCR4*) and empty spiracles homolog 2, Drosophila (*EMX2*) genes were down-regulated in both suicide groups compared to the non-suicide groups. As negative controls, the normalization control probe set of 100 genes were tested by the same ANOVA model, and no genes met our statistical criteria (Fold Change ≥ |1.3| and FDR < 0.1) between the suicide group vs. non-suicide group within bipolar or schizophrenia diagnostic categories.

**Table 3:** Summary of differentially expressed genes between the suicide completer group vs. non-suicide group in schizophrenia with fold change relative to non-suicide group.

**Gene symbol**	**Description**	**FC**^1^	**FDR**^2^	**P/A call**^3^
**GJA1**	gap junction protein, alpha 1, 43 kDa (connexin 43)	-2.0	0.02	P
**MT1X**	metallothionein 1X	-1.9	0.04	P
**MT1M**	metallothionein 1M	-1.9	0.06	P
**AGXT2L1**	alanine-glyoxylate aminotransferase 2-like 1	-1.9	0.03	P
**SDC4**	syndecan 4 (amphiglycan, ryudocan)	-1.7	0.01	P
**SOX9**	SRY (sex determining region Y)-box 9	-1.7	0.02	P
**NTRK2**	neurotrophic tyrosine kinase, receptor, type 2	-1.7	0.02	P
**HSPB1**	heat shock 27 kDa protein 1	-1.7	0.04	P
**EIF5A**	eukaryotic translation initiation factor 5A	-1.7	0.06	P
**EFEMP1**	EGF-containing fibulin-like extracellular matrix protein 1	-1.6	0.04	P
**ID4**	inhibitor of DNA binding 4, dominant negative helix-loop-helix protein	-1.6	0.02	P
**APOE**	apolipoprotein E	-1.6	0.03	P
**PLSCR4**	phospholipid scramblase 4	-1.6	0.03	P
**AGT**	angiotensinogen (serpin peptidase inhibitor, clade A, member 8)	-1.6	0.02	P
**TUBB2B**	tubulin, beta 2B	-1.5	0.04	P
**MT1E**	metallothionein 1E (functional)	-1.5	0.03	P
**FAM107A**	family with sequence similarity 107, member A	-1.5	0.03	P
**SLC4A4**	solute carrier family 4, sodium bicarbonate cotransporter, member 4	-1.5	0.05	P
**GLUL**	glutamate-ammonia ligase (glutamine synthetase)	-1.5	0.04	P
**ALDH1L1**	aldehyde dehydrogenase 1 family, member L1	-1.5	0.02	P
**DDIT4**	DNA-damage-inducible transcript 4	-1.5	0.04	P
**MT1G**	metallothionein 1G	-1.5	0.04	P
**SLC1A3**	solute carrier family 1 (glial high affinity glutamate transporter), member 3	-1.5	0.08	P
**PPAP2B**	phosphatidic acid phosphatase type 2B	-1.5	0.05	P
**EMX2**	empty spiracles homolog 2 (Drosophila)	-1.5	0.05	P
**TP53BP2**	tumor protein p53 binding protein, 2	-1.5	0.03	P
**MT1H**	metallothionein 1H	-1.5	0.02	P
**APOLD1**	apolipoprotein L domain containing 1	-1.4	0.08	P
**FGFR3**	fibroblast growth factor receptor 3 (achondroplasia, thanatophoric dwarfism)	-1.4	0.03	P
**FXYD1**	FXYD domain containing ion transport regulator 1 (phospholemman)	-1.4	0.00	P
**NOTCH2**	Notch homolog 2 (Drosophila)	-1.4	0.03	P
**METTL7A**	methyltransferase like 7A	-1.4	0.05	P
**SLC14A1**	solute carrier family 14 (urea transporter), member 1 (Kidd blood group)	-1.4	0.06	P
**BBOX1**	butyrobetaine (gamma), 2-oxoglutarate dioxygenase 1	-1.4	0.03	P
**MT1F**	metallothionein 1F (functional)	-1.4	0.05	P
**EPHX1**	epoxide hydrolase 1, microsomal (xenobiotic)	-1.4	0.02	P
**SLC7A11**	solute carrier family 7, (cationic amino acid transporter, y+ system) member 11	-1.4	0.05	P
**TPD52L1**	tumor protein D52-like 1	-1.4	0.02	P
**RHOBTB3**	Rho-related BTB domain containing 3	-1.4	0.05	P
**VIL2**	villin 2 (ezrin)	-1.4	0.03	P
**NDRG2**	NDRG family member 2	-1.4	0.06	P
**CLDN10**	claudin 10	-1.4	0.06	P
**NTSR2**	neurotensin receptor 2	-1.4	0.03	A
**ITPKB**	inositol 1,4,5-trisphosphate 3-kinase B	-1.4	0.10	P
**SDC2**	syndecan 2 (heparan sulfate proteoglycan 1, cell surface-associated, fibroglycan)	-1.4	0.03	P
**IL17RB**	interleukin 17 receptor B	-1.3	0.03	P
**SPON1**	spondin 1, extracellular matrix protein	-1.3	0.02	P
**LOC645745 **	metallothionein 1H-like protein	-1.3	0.03	P
**ITGB4**	integrin, beta 4	-1.3	0.03	P
**MLC1**	megalencephalic leukoencephalopathy with subcortical cysts 1	-1.3	0.05	P
**ATP1A2**	ATPase, Na+/K+ transporting, alpha 2 (+) polypeptide	-1.3	0.05	P
**RAB31**	RAB31, member RAS oncogene family	-1.3	0.05	P
**ATP1B2**	ATPase, Na+/K+ transporting, beta 2 polypeptide	-1.3	0.05	P
**AHCYL1**	S-adenosylhomocysteine hydrolase-like 1	-1.3	0.03	P
**CX3CR1**	chemokine (C-X3-C motif) receptor 1	2.1	0.02	P
**C3 **	complement component 3	1.6	0.02	P
**CRH**	corticotropin releasing hormone	1.6	0.05	P
**ABCG2**	ATP-binding cassette, sub-family G (WHITE), member 2	1.5	0.03	P
**DUSP6**	dual specificity phosphatase 6	1.5	0.04	P
**NPY**	neuropeptide Y	1.4	0.03	P
**SLC25A23**	solute carrier family 25 (mitochondrial carrier; phosphate carrier), member 23	1.4	0.03	A
**LAPTM5**	lysosomal associated multispanning membrane protein 5	1.4	0.03	P
**TNFSF10**	tumor necrosis factor (ligand) superfamily, member 10	1.4	0.01	P
**TYROBP**	TYRO protein tyrosine kinase binding protein	1.4	0.03	P
**CD74**	CD74 molecule, major histocompatibility complex, class II invariant chain	1.4	0.03	P
**P2RY13**	purinergic receptor P2Y, G-protein coupled, 13	1.3	0.02	A
**CSF1R**	colony stimulating factor 1 receptor	1.3	0.03	P
**HLA-DRA**	major histocompatibility complex, class II, DR alpha	1.3	0.04	A
**HLA-DPA1**	major histocompatibility complex, class II, DP alpha 1	1.3	0.05	P
**A2M**	alpha-2-macroglobulin	1.3	0.03	P

^1^FC represents ratio of geometric means between suicides vs. non-suicide.

^2 ^P-values were adjusted by FDR.

^3^Absent (A) or Present (P) detection call by MAS5 statistical algorithm

Real time-PCR (RT-PCR) tested the validity of these two shared, differentially expressed genes in the available subset of the microarrayed samples. In the schizophrenia suicide group, the *EMX2 *and *PLSCR4 *expression levels were significantly down-regulated by comparison of mean expression levels (*EMX2 *t(9) = 2.42, p = 0.02; *PLSCR4 *t(18) = 3.77, p = 0.0005) in the suicide group compared to the non-suicide group (Fig 1B). The estimated fold changes in the suicide group were -1.51 for *EMX2 *and -2.16 for *PLSCR4 *relative to the non-suicide group. These differences were consistent with our microarray data. However, in bipolar disorder, these two genes could not be validated in a subset of tissues available from the original microarray study patients (Fig. 1B). These results highlight the overall findings that few common differentially genes for suicide vs. non-suicide exist between diagnostic groups.

### Biological process in the differentially expressed genes

**Figure 2:**
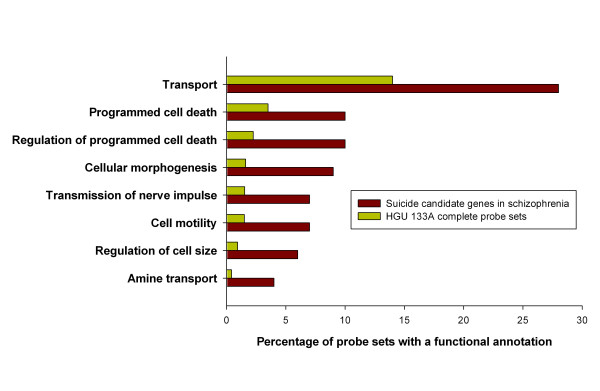
Distribution of gene ontology groups for biological process at level 4 of differentially expressed genes between suicide vs. non-suicide in schizophrenia cohorts.

Functional annotation of the differentially expressed genes by Gene Ontology indicated that 9 biological processes were significantly overrepresented (at level 4; P < 0.05) among the suicide candidate genes in the schizophrenia cohort (Fig. 2). The transport genes were the most commonly over-represented biological process in our suicide candidate gene list for schizophrenia, including the glial, high affinity, glutamate transporter (*SLC1A3*). In contrast, no significantly over-represented biological process group emerged with suicide candidate genes in the bipolar disorder group.

**Figure 3:**
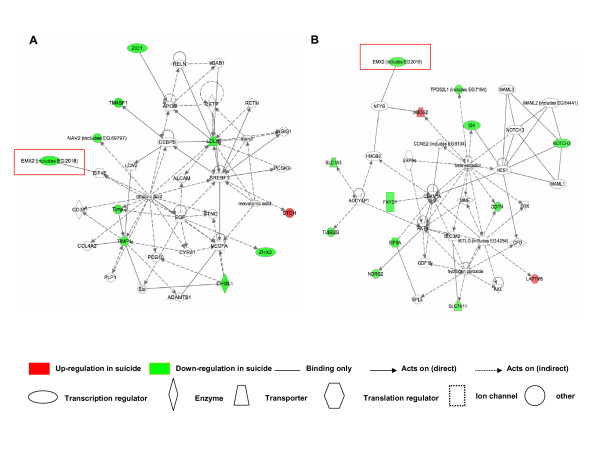
Ingenuity pathway analysis of *EMX2 *and differentially expressed suicide candidate genes in bipolar disorder (A) and schizophrenia (B).

Additionally, we analyzed the list of differentially expressed genes for each diagnosis by the Ingenuity Pathways Analysis (IPA) software to identify biological pathways and networks. We identified distinct signaling networks from suicide candidate genes that included the *EMX2 *gene in both disorders (Fig. 3). In bipolar disorder, the pathway perspective suggested a signaling network related to both cellular movement and cell to cell signaling, with interactions encompassing 10 differentially expressed, suicide candidate genes (Fig. 3A). By contrast, in schizophrenia patients, the differentially expressed genes were related in a cell death signaling network (Fig. 3B).

## Discussion

In this re-analysis study, we explored suicide candidate genes associated with bipolar disorder and schizophrenia using an unbiased genome-wide expression profiling strategy. To identify suicide specific effects on the expression level of genes, we compared a suicide completers group to a non-suicide group within the same diagnostic category. The most important finding of this study is the identification of suicide candidate gene lists for bipolar disorder and schizophrenia with only two differentially expressed (suicide vs. non-suicide) genes in both bipolar and schizophrenia cohorts namely *PLSCR4 *and *EMX2 *by microarray analysis. The overlap of the two gene lists is small, suggesting few common, shared genes. For schizophrenia, but not bipolar disorder, the differential expression of *PLSCR4 *and *EMX2 *was confirmed by RT-PCR. The Phospholipid scramblase (PLSCR) is an integral membrane protein that catalyzes Ca^2+^-induced bidirectional movement of phospholipids [16]. Four isoforms have been cloned, and *PLSCR4 *was the major isoform expressed in the brain [17]. However, the biological role of the PLSCR4 remains unknown. While speculative, the changes in phospholipids membrane composition might have pleiotropic effects as evidence suggests that membrane composition can change G protein coupled receptors' functioning and downstream effector signalling [18,19] as well as voltage-dependent K+ channels [20]. EMX2 is a homeodomain containing transcription factor, which plays a crucial role in forebrain patterning and development in mouse models [21]. This finding suggests a possible neurodevelopmental process whereby variation in forebrain development may be a risk factor for suicide completion behaviour associated with schizophrenia. Of note, these differentially expressed genes are neural correlates of suicide and not necessarily causal. They could be epiphenomenon. The questions remain of 1) how these genes function to influence suicide and 2) what intermediate phenotype would be appropriate to demonstrate their possibly causal role.

Microarray studies with small sample sizes result in low statistical power and its attendant "noise discovery". This analysis and post-mortem sample size with replicates is noteworthy for an adequately powered sample to detect 1.3 fold changes, improving sensitivity, reliability, and signal to noise issues.

Previous smaller microarray studies have suggested that GABA_A _receptor subunits and glutamate-related genes were differentially expressed in bipolar disorder and schizophrenia as well as in suicide completers associated with these disorders [8,10]. We identified the up-regulation of gamma-amino butyric acid A receptor, α5 (*GABRA5*) subunit gene in suicide associated with bipolar disorder, confirming a previous report [10]. The expression levels of two glutamate-related genes, Glutamate-ammonia ligase (*GLUL*) and glial high affinity glutamate transporter member 3 (*SLC1A3*) were decreased in suicide completers with schizophrenia. The serotonergic and noradrenergic systems have been suggested to be associated with suicide [22]. However, no genes related to these two neurotransmitter systems were identified, consistent with a previous report [23]. This negative finding may suggest indirect effects on these neurotransmitter systems.

Genetic linkage studies have identified several loci associated with suicidal behaviors in bipolar disorders. Significant and suggestive linkages for suicide were mapped on chromosome 2, 5, and 10 from 162 bipolar pedigrees [24]. Among this study's suicide candidate genes associated with bipolar disorder, the tripartite motif-containing 23 (*TRIM23*) gene is located close to the significantly linked D5S1725 marker on chromosome 5. Another large scale genetic linkage study for bipolar disorder using 1060 individuals identified linkage on chromosome 10q25.3 for suicide attempts [25]. The microarray differentially expressed candidate gene *EMX2 *is included in this region of interest. Therefore, these two genes may be apt for future genetic association studies for suicide associated with bipolar disorder, proving causation.

While this reanalysis study has the strengths of a larger sample size, independent replicates, and well characterized patient samples from specific areas of cortex, the finding should be interpreted cautiously as this study has some limitations. First, the mixed cellular nature of the brain samples might lower sensitivity due to dilutional effects as opposed to pure neuronal cells of a specific cortical layer [12]. In general, most microarray studies with post-mortem brain tissues find fold changes of less than 2 fold, including this study. Second, although smoking, alcohol, and drug abuse were measured as confounding factors, all possible unmeasured, confounding variables for suicide cannot be formally excluded, such as severity of illness (especially the last episode for suicide completers), personality traits, hopelessness, agitation, depressive symptoms, and stress. Third, these findings are correlational and not causal. Fourth, these gene lists should be considered as provisional until confirmed by replications in independent sets of biological samples.

## Conclusion

By reanalyzing a large microarray dataset, a list of differentially expressed candidate genes for suicide within bipolar disorder or within schizophrenia have been identified. The overlap of genes in common among these two gene lists is small, with a larger number of disorder specific genes being found. This finding suggests that disorder-specific pathways predominate over common pathways at the molecular level. Two novel candidate genes, *PLSCR4 *and *EMX2*, were confirmed as differentially expressed in schizophrenia between suicide completers vs. non-suicide groups.

## Methods

### Microarray data and Patient Samples

The brain tissues were meticulously collected in a standardized manner via pathologists in the offices of the Medical Examiner in several states with the families' permission under the aegis of the Stanley Foundation Brain Collection (Array Collection plus Consortium Collection) [26]. The selection of specimens, clinical information, diagnoses of patients, and processing of tissues were conducted by Stanley Foundation Consortium as described previously [26]. Gene expression profiling utilized post-mortem prefrontal cortex (Brodmann's Area 46/10) mRNA and Affymetrix Human Genome U133 Set A (HGU133A) using standardized techniques as described [26,27]. The prefrontal cortex was selected as the region of interest due to its role in executive functioning, impulsivity ("lack of premeditation"), and decision making. Disadvantageous decision making and impulsivity have been found to increase the risk of suicide [28,29].

The Stanley Foundation's microarray database is an anonymous, de-identified dataset without any protected health information. Patients' demographic variables used in this study are listed in Table 1.

The robust multi-array averages (RMA)-normalized microarray data from four independent studies were downloaded from the SMRIDB. Microarray data from the same platform, Affymetrix Human Genome U133 Set A (HGU133A), were used to avoid platform-to-platform variation. The platform contains 22,215 probe sets. Quality control analyses for each chip were described previously [15]

For the bipolar disorder cohort, the total dataset consisted of 49 suicide completers' gene chips and 58 non-suicide gene chips, while for schizophrenia cohort, the total dataset consisted of 22 suicide completer gene chips and 89 non-suicide chips. Among 45 bipolar samples, there were 22 suicide cases, and 23 non-suicide cases. Among 45 schizophrenia patients, there were 10 suicide cases, and 35 non-suicide cases. Two to three microarray chip datasets were generated from the each patient's sample. These repeated microarray data from each patient were treated as technical replicates.

### Statistical analysis of microarray data

Microarray data was analyzed by a statistical method described previously with slight modifications [26]. Briefly the following steps were followed within each diagnostic group (see Table 1). First, the differentially expressed genes between suicide completers vs. non-suicide groups were filtered by average fold change (FC ≥ |1.3|) using the BRB-array tool[30] without covariates. Second, the influence of continuous demographic variables (such as age, post-mortem interval (PMI) and brain pH) with the nominal variable suicide was tested using ANOVA. Then, categorical variables such as sex, smoking, alcohol and drug abuse were tested using chi square tests of association (Statview software SAS, Cary, NC). In addition, correlation analyses of the demographic factors with expression levels of the differentially expressed probe sets from step 1 were performed. Continuous variables were analyzed by Spearman's rank correlation and categorical variables were tested by ANOVA. P-values were adjusted by False Discovery Rate (FDR) in both tests [31]. Third, significant confounding factors were tested as possible covariates for ANCOVA model inclusion with the following criteria: The variable was required to show both 1) significant association with suicide as well as 2) significant correlation with expression levels of the differentially expressed genes. However, no variables met the criteria in both disorder groups. Therefore, no covariates were used in the omnibus ANOVA, using the factor as suicide vs. non-suicide. As an exploratory analysis, a more liberal FDR P-value (< 0.1) of significance was selected for expression level differences as previously described [32,33], using the BRB array software tool's FDR default setting. For negative controls, we performed statistical analysis with the HGU133A normalization control probe sets using the same ANCOVA models, ensuring the adjustment did not produce "noise" or aberrant false positives. The Microarray Suite, version 5.0 (MAS5) software was used to filter genes with low expression levels as either present or absent, applying the detection call statistical algorithm. This algorithm suggests whether a gene is present or absent.

A power analysis estimated the sample sizes for detection of a 1.3 fold change in a gene with a significance criterion of P-value = 0.001 and a power of 0.90 using a previously described method [13]. This analysis estimated a minimum sample size of 27 cases per group for comparing suicide completers vs. non-suicide groups within bipolar disorder and 21 samples per group for comparing suicide completers vs. non-suicide completers within schizophrenia.

### Real-time quantitative PCR

Total RNA from the dorsolateral prefrontal cortex (Brodmann area 46) of the Array Collection was used for this experiment. Complementary DNA was synthesized from DNA-free RNA with a random hexamer primer and Superscript III First-Strand Synthesis System according to the manufacturer's protocol (Invitrogen). Using a 384-well format with the Prism7900HT real-time detector (ABI), 2 μl aliquots of (10×) QuantiTect Primer Assay (validated primers to the specific gene of interest; Qiagen), 10 μl (2×) QuantiTect SYBR PCR Master mix (Qiagen), and 8 μl cDNA were mixed together for 20 μl total reaction volume and pipetted into single wells of the 384 PCR plate. Amplification conditions were: (1) 1 cycle for 2 min at 50°C, (2) 1 cycle for 15 min at 95°C, and (3) 45 cycles for 15 s at 94°C, 30 s at 60°C and 30 s at 72°C and fluorescence was measured during the 72°C step for each cycle as recommended by the manufacturer. The β-2 microglobulin (B2M) was chosen as an endogenous control for the normalization of target genes as it was consistently expressed in microarray samples. A total of 32 samples per each disorder were used for this experiment and run in duplicate. In the bipolar disorder cohort, there were 14 suicides and 18 non-suicide cases. In schizophrenia cohort, there were 5 suicides and 27 non-suicide cases. These samples were matched by age, race, gender, PMI, brain pH, side of the brain and quality of RNA. Reactions were quantified by the comparative Ct method using SDS2.2 software (ABI). This RT-PCR data was also statistically analyzed by the amplification plot method using the Data Analysis for Real Time PCR (DART-PCR) approach [34]. This method identified outliers in amplification efficiency by ANOVA and calculated mean expression levels. Statistical differences in expression levels between groups, namely suicide completers vs. non-suicides within a diagnostic group, were tested by one-tail, t-test with unequal variances (Microsoft Excel) as described [35,36]. To estimate average fold changes between groups, the mean expression values from the DART-PCR approach were used. The alternative 2(-Delta Delta Ct) method [37] for estimating fold change (using all the data without exclusions) verified this fold change estimate. As both methods gave similar estimates, only the DART-PCR approach estimates from mean expression levels were reported.

### Functional annotation

The differentially expressed genes were functionally annotated using the DAVID integrated database query tool[38] and by the over-representational analysis method [39]. Functional annotations were based on biological process of Gene Ontology (GO) Consortium[40] at level 4. P-values less than 0.05 were considered significant.

### Pathway Analysis

Biologically relevant networks were drawn from the lists of genes that were differentially expressed in bipolar disorder and schizophrenia. This data was generated through the use of Ingenuity Pathways Analysis (IPA) [41], a web-delivered application that enables the visualization and analysis of biologically relevant networks to discover, visualize, and explore relevant networks. Expression data sets containing gene identifiers (Affymetrix probe set ID) and their corresponding expression values as fold changes were uploaded as a tab-delimited text file. Each gene identifier was mapped to its corresponding gene object in the Ingenuity Pathways Knowledge Base. These genes, called Focus Genes, were then used as the starting point for generating biological networks. To start building networks, the application program queries the Ingenuity Pathways Knowledge Base for interactions between Focus Genes and all other gene objects stored in the knowledge base, and generates a set of networks. The program then computes a score for each network according to the fit of the network to the set of focus genes. The score indicates the likelihood of the Focus Genes in a given network being found together due to random chance. A score of greater than 2 indicates that there is a less than 1 in 100 chance that the Focus Genes were assembled randomly into a network due to random chance. The scores of the networks generated from the lists of differentially expressed genes were 24 for Bipolar disorder (Fig 3A) and 40 for Schizophrenia (Fig 3B).

## Authors' contributions

SK performed the analysis of the microarray data. HG supervised the study. SK and HG wrote the manuscript. KC performed qRT-PCR validation experiment and SK performed analysis of the data. AFB provided important discussions. All authors read and approved of the final version of manuscript.
